# Valine-Curcumin Improves Growth, Intestinal Immunity, and Microbiota in Largemouth Bass (*Micropterus salmoides*)

**DOI:** 10.3390/ani16132032

**Published:** 2026-07-02

**Authors:** Jing Ni, Hejian Xiong, Ruifang Wang, Yuanhong Xie, Lixing Huang, Ying Ma, Chuanbo He

**Affiliations:** 1 State Key Laboratory of Mariculture Breeding, Fisheries College of Jimei University, Xiamen 361021, China; nijing9377@163.com (J.N.); lixinghuang@jmu.edu.cn (L.H.); 2 College of Ocean Food and Biological Engineering, Jimei University, Xiamen 361021, China; hjxiong@jmu.edu.cn (H.X.); wangrf@jmu.edu.cn (R.W.); xieyuanhongjd@126.com (Y.X.)

**Keywords:** valine-curcumin, largemouth bass, growth performance, antioxidant activity, immune regulation, gut microbiota

## Abstract

Curcumin (Cur), a polyphenol with excellent antioxidant and anti-inflammatory properties, is widely used in aquaculture. However, its low water solubility limits bioavailability. This study first investigated the effects of a highly water-soluble and bioavailable valine-curcumin (Val-Cur) on growth performance, antioxidant capacity, inflammatory factor expression and gut microbiota in juvenile largemouth bass. A total of 450 healthy largemouth bass (initial weight 12.00 ± 0.45 g per fish) were randomly divided into six groups: the basal diet group (CK), the group supplemented with 60 mg/kg of curcumin (Cur), and groups that were fed with 15, 30, 60, and 120 mg/kg of Val-Cur. Each group had three replicates (25 fish per replicate). After 8 weeks, compared with both the CK and Cur groups, 30–60 mg/kg Val-Cur displayed significantly increased growth rates, specific growth rates, and protein conversion efficiency, and significantly decreased the feed conversion ratio. Quadratic regression analysis indicated that the optimal supplementation level was approximately 51.62 mg/kg Val-Cur. The Cur and Val-Cur between 30 and 60 mg/kg groups also displayed significantly improved serum biochemical indicators (↑HDL-C, ALB, LZM, CAT and SOD, and ↓MDA). These groups also promoted the expression of intestinal anti-inflammatory factors (*TGF-β1* and *IL-10*) and physical barrier genes (*Claudin1*, *Occludin* and *Claudin4*). In terms of regulating the intestinal microbiota, both Cur and Val-Cur significantly reduced the bacterial diversity (↓Sobs, Chao1 and PD indices) and increased the evenness of bacterial distribution (↑Simpson, Shannon and Pielou indices). In addition, the abundance of some potential pathogens (e.g., *Plesiomonas*, *Plesiomonas shigelloides*, and *Pseudomonas fluorescens*) significantly decreased, while the abundance of beneficial bacteria (e.g., *Faecalibaculum*, *Faecalibaculum rodentium* and *Lactobacillus murinus*) significantly increased. Pearson’s correlation analysis showed that the increase in the abundance of beneficial bacteria was positively correlated with improvement in growth performance, serum biochemical and antioxidant capacity indicators. In summary, Val-Cur exerted superior biological effects at lower dietary inclusion levels than Cur. This study laid a theoretical foundation for elucidating the mechanism of Val-Cur in improving fish immunity and promoting the application of water-soluble curcumin in aquaculture.

## Simple Summary

Curcumin holds great potential as a natural immunostimulant in animal nutrition, yet its practical application is severely constrained by low water solubility and poor bioavailability. We previously synthesized a highly water-soluble valine-curcumin conjugate (Val-Cur). This study now presents the first comprehensive in vivo evaluation of Val-Cur in largemouth bass, systematically revealing its enhanced efficacy and underlying physiological mechanisms. We found that dietary Val-Cur at doses as low as 15–30 mg/kg outperforms native curcumin (60 mg/kg) across multiple key metrics: it more effectively promotes growth, improves serum lipid profiles, and strengthens systemic antioxidant and anti-inflammatory responses. These benefits are achieved through a coordinated mechanism that includes reinforcing intestinal barrier integrity, modulating apoptosis-related gene expression, and beneficially reshaping the gut microbiota—specifically by inhibiting potential pathogens while enriching beneficial, short-chain fatty acid-producing bacteria. The poor water solubility of curcumin is a major bottleneck in animal nutrition. Our findings demonstrate that an amino acid conjugation strategy effectively reduces the effective dose while surpassing native curcumin in efficacy, representing an important step for functional feed development.

## 1. Introduction

Under intensive aquaculture systems, factors such as high-density farming, overfeeding, and the deterioration of the breeding environment lead to the frequent occurrence of aquaculture diseases, which cause oxidative stress in fish, inhibit the growth of fish and even lead to death [1]. Concurrently, the abuse of antibiotics increases the risk of bacterial resistance and food safety, which seriously restricts the sustainable development of aquaculture [2]. Several plant-derived natural active substances have been used as immunostimulants and growth promoters to replace antibiotics in animal feed. Among them, curcumin (Cur) has considerable potential in the field of aquaculture because of its successful application over a relatively wide dietary supplementation range (50 mg/kg to 2 g/kg) and various biological activities. Studies have shown that dietary Cur supplementation has the effects of promoting growth, antioxidant activity, and immune regulation, preventing disease and regulating the gut microbiota in aquatic animals [3].

The appropriate additive dosage of Cur in aquafeed varies by species. Supplementation of 400 mg/kg Cur has no significant effect on the growth of rainbow trout [4], but 1000 mg/kg Cur improved the feed efficiency of carp [5]. Supplementation of Cur at 100 mg/kg to 5 g/kg improved the hematological parameters (increasing red and white blood cell counts and hematocrit levels), significantly reduced MDA levels in the kidney and liver, and improved the antioxidant capacity of carp (*Cyprinus carpio*) [6]. Supplementation of 150 mg/kg Cur significantly improved the resistance of silver catfish to *Streptococcus agalactiae* [7], and 20 g/kg Cur significantly increased the relative survival rate of rainbow trout infected with *Aeromonas salmonicida* [4]. Zou et al. found that 100 mg/kg Cur decreased the alpha diversity of gut microbiota and increased the abundance of *Mycoplasma*, *Psychrobacter* and *Vibrio* in abalone intestines [8]. At present, most studies involve the addition of extremely high doses of Cur [3], which are costly and produce inconsistent effects, and the effective doses vary greatly (from 100 mg/kg to 20 g/kg). This may be due to the low solubility, poor stability, rapid metabolism in vivo and low bioavailability of Cur [9].

In previous work, our research team successfully synthesized a highly water-soluble valine-curcumin conjugate (Val-Cur) via an amino acid conjugation, which increased its solubility by 9000-fold (from 1.9 × 10^−4^ mg/mL to 1.78 mg/mL). Studies have shown that Val-Cur exhibits strong antibacterial activity in vitro, alongside diverse in vivo biological functions, including promoting fish growth and modulating gut microbiota structure [10]. The largemouth bass (*Micropterus salmoides*) is an important commercially farmed freshwater species in China, valued for its rapid growth rate, broad temperature tolerance, palatable flesh, and absence of intermuscular bones. According to the 2024 “China Fisheries Statistical Yearbook”, its total aquaculture production has been steadily increasing and has exceeded 888,000 metric tons [11]. Cur, a functional feed additive with multiple pharmacological properties, has been experimentally incorporated into largemouth bass feed with promising results. For instance, dietary supplementation with Cur (60 mg/kg) has been shown to enhance the non-specific immune response and antioxidant capacity [12]. It has also been demonstrated to significantly mitigate pesticide-induced (e.g., chlorpyrifos) oxidative stress, cell apoptosis and immune dysfunction, thereby reducing the damage caused by such contaminants [13]. Preliminary research by our team indicates that Val-Cur exhibits higher in vivo bioavailability and a greater potential to protect largemouth bass against pathogens than native Cur [14]. However, whether the improved water solubility and bioavailability of Val-Cur translate into enhanced immunomodulatory effects compared to Cur in largemouth bass remains unreported. This study aims to systematically compare the effects of Val-Cur and Cur on the antioxidant response, immune response, and intestinal microbiota of juvenile largemouth bass. The findings are expected to elucidate the mechanism by which water-soluble curcumin enhances fish immunity, thereby facilitating its further application in sustainable aquaculture.

## 2. Materials and Methods

### 2.1. Feeding Trial

A total of 500 healthy juvenile largemouth bass, with an average body weight of 12.00 ± 0.45 g/fish, were obtained from Zhaoan Minxi Fish farm (Zhaoan, Fujian, China). The fish were acclimated for two weeks under controlled conditions: water temperature 26–28 °C, pH 7.2–7.5, dissolved oxygen 8 mg/L, ammonia nitrogen < 0.1 mg/L, and nitrite < 0.005 mg/L. After acclimation, the fish were fasted for 24 h. Subsequently, 450 healthy fish of consistent size were selected and randomly distributed into six experimental groups, with three replicates per group (each consisting of three 400 L culture buckets containing 25 fish). The six experimental diets were as follows: a basal diet (control group, CK), a basal diet supplemented with 60 mg/kg Cur (Cur group), and basal diets supplemented with 15, 30, 60, or 120 mg/kg Val-Cur (designated Val-Cur15, Val-Cur30, Val-Cur60, and Val-Cur120 groups, respectively). Supplementation levels were expressed as the actual amounts added to the diets (mg/kg diet) rather than curcumin-equivalent concentrations. Cur (95% purity) was purchased from Aladdin Co., Ltd. (Shanghai, China), and Val-Cur was prepared as described previously [10]. The formulation of the basal diet has been reported previously, containing 47.84% crude protein, 11.85% crude lipids, and 12.18% total ash [14]. The 8-week feeding trial began with feeding at 2% of body weight, adjusting thereafter to satiation, and daily feed intake was recorded. No significant differences in feed intake among treatment groups were observed. Water quality and management conditions were consistent with the acclimation period. The animal study was reviewed and approved by the Animal Ethics Committee of Jimei University (Acceptance no. JMULAC201159).

### 2.2. Sample Collection

After the 8-week feeding trial, fish were fasted for 24 h prior to sampling. From each replicate tank, fifteen fish were randomly selected and anesthetized in 200 mg/L MS-222 (Sigma-Aldrich, Burlington, MA, USA) for 10 min. After anesthesia, the body surface of each fish was wiped dry to allow for the accurate measurement of the body length and weight. Blood samples were drawn from the caudal vein using sterile 1 mL syringes and allowed to clot at 4 °C overnight, and then centrifuged at 3000 r/min for 10 min at 4 °C. The resulting serum supernatant was aliquoted into 1.5 mL tubes and stored at −80 °C for subsequent biochemical analysis. Intestines were aseptically dissected on ice from a total of 30 fish per group (10 per replicate). Subsamples were allocated for subsequent analyses. For gene expression analysis, intestinal samples from three fish per replicate were collected, and total RNA was extracted individually from each fish for qPCR analysis (*n* = 9 per group). For intestinal microbiota analysis, intestinal samples from four fish per treatment group were collected for sequencing (*n* = 4 per group). No samples were pooled prior to analysis. All samples were immediately flash-frozen in liquid nitrogen and stored at −80 °C. Additionally, intestines from two separate fish per replicate (totaling 6 per group) were rinsed with 0.8% physiological saline, blotted dry on filter paper, flash-frozen, and stored at −80 °C for physiological and biochemical assays. The midgut tissues from another two fish per replicate (totaling 6 per group) were fixed in Bouin’s solution for subsequent histological examination. The calculation formulas for growth performance indicators are as follows:

Survival rate, SR (%) = 100 × Nf/Ni

Weight gain rate, WGR (%) = 100 × (Wf − Wi)/Wi

Specific growth rate, SGR (%/d) = 100 × (InWf − InWi)/t

Feed conversion rate, FCR (%) = FC/(Wf − Wi)

Protein efficiency rate, PER (%) = 100 × (Wf − Wi)/(FC × Pd)

Condition factor, CF (g/cm^3^) = 100 × Wf/L3

where Wi is the initial body weight, Wf is the final body weight, FC is the average feed consumption, t is the number of feeding days, Pd is the crude protein content of the feed; L is the final body length of fish, Ni is the initial number of fish, and Nf is the final number of fish.

### 2.3. Analysis of Serum Biochemical Parameters

The measured serum biochemical parameters included albumin (ALB) content, lysozyme (LZM) activity and the concentrations of triglyceride (TG), total cholesterol (TC), high-density lipoprotein cholesterol (HDL-C), and low-density lipoprotein cholesterol (LDL-C). All parameters were quantified using commercial assay kits (purchased from the Nanjing Jiancheng Bioengineering Institute, Nanjing, China) strictly in accordance with the manufacturer’s instructions.

### 2.4. Analysis of Intestinal Antioxidant Enzyme Activities

Intestinal tissue samples were homogenized in a pre-chilled automated cryogenic grinder (JXFSTPRP-24L, Shanghai Jingxin Industrial Development Co., Ltd., Shanghai, China) with a 10-fold (*w*/*v*) volume of normal saline solution (0.7% NaCl). The homogenates were then centrifuged at 3000 g for 10 min. The resulting supernatant was collected and stored at 4 °C for subsequent analysis. The activities of superoxide dismutase (SOD), catalase (CAT), glutathione peroxidase (GSH-Px), as well as the total antioxidant capacity (T-AOC) and malondialdehyde (MDA) content were measured using commercial assay kits (Nanjing Jiancheng Bioengineering Institute, Nanjing, China). All procedures were performed in accordance with the manufacturer’s protocols.

### 2.5. Intestinal Histopathological Examination

The fixed intestinal tissues were embedded in paraffin and sectioned into 5 µm thick slices using a microtome. The sections were then deparaffinized with xylene and rehydrated through a descending graded ethanol series (from 100% to 75%). After staining with hematoxylin and eosin (H&E), the sections were dehydrated through an ascending ethanol series and permanently mounted under a coverslip. Finally, the slides were observed, measured, and photographed using an upright fluorescence microscope (Nikon Eclipse Ci, Tokyo, Japan).

### 2.6. Real-Time Quantitative PCR (RT-qPCR)

The relative mRNA expression levels of genes related to antioxidant enzymes, inflammatory factors, physical barriers, and apoptosis in the intestine were determined by RT-qPCR. Total RNA was first extracted from intestinal tissues using a Trizol RNA kit (TransGen, Beijing, China). The integrity of the RNA was verified by 1.2% denaturing agarose gel electrophoresis, while its purity and concentration were measured with a NanoDrop 2000 spectrophotometer (Thermo Fisher, Waltham, MA, USA). Only RNA samples showing intact rRNA bands and acceptable purity (A260/A280 ratio between 1.8 and 2.0) were used for subsequent cDNA synthesis. Subsequently, complementary DNA (cDNA) was synthesized from the qualified RNA using TransScript All-in-One First-Strand cDNA Synthesis SuperMix (TransGen, Beijing, China). Then, qRT-PCR was performed on a QuantStudio 6 Flex Real-Time PCR system (Thermo fisher, Waltham, MA, USA). Each 10 μL reaction mixture contained 5 μL of SYBR Green qPCR Master Mix (TransGen, Beijing, China), 0.25 μL of forward primer, 0.25 μL of reverse primer, and 4.5 μL of cDNA template (diluted to an appropriate concentration with RNase free water). The relative expression levels of target genes were calculated using the 2^−ΔΔCt^ method [15]. All primers, including those for the reference gene (β-actin) and antioxidant-related genes, were designed and synthesized by Shanghai Sangon Biotech Co., Ltd. (Shanghai, China), and their sequences are listed in Table 1.

### 2.7. 16S rDNA Sequencing and Bioinformatic Analysis

Total DNA was extracted from frozen intestinal tissues using the MolPure^®^ Stool DNA Kit (Yeasen, Shanghai, China). The purity and concentration of the DNA were assessed with a NanoDrop 2000 (Thermo Fisher Scientific, Waltham, MA, USA). The V3–V4 region of the bacterial 16S rRNA gene was amplified by PCR. Amplicons were sequenced on an Illumina NovaSeq 6000 platform at Guangzhou Gidion Bio-Technology Co., Ltd. (Gunagzhou, China). The raw paired-end reads were merged using FLASH and quality-filtered with UPARSE (version 7.1). High-quality sequences were clustered into operational taxonomic units (OTUs) at a 97% similarity threshold. Taxonomic annotation of representative OTU sequences was performed using the Ribosomal Database Project (RDP) classifier. Alpha diversity indices (Chao1, Shannon and Simpson) were calculated using Mothur software (version 1.48.5). Beta diversity was assessed via non-metric multidimensional scaling (NMDS) based on Bray–Curtis distances. Differences in microbial community composition among groups were further evaluated using permutational multivariate analysis of variance (PERMANOVA) with 999 permutations, and the corresponding R^2^ and *p* values were reported.

### 2.8. Statistical Analysis

Data are presented as the mean ± standard deviation (SD). Statistical analyses were performed using SPSS 22.0 (IBM, Armonk, NY, USA). Prior to one-way analysis of variance (ANOVA), normality of residuals and homogeneity of variance were assessed using the Shapiro–Wilk test and Levene’s test, respectively. Differences among groups were evaluated by one-way analysis of variance (ANOVA) followed by Duncan’s test. A Pearson correlation analysis was conducted to assess the relationships between significantly altered gut microbiota, growth performance parameters, and serum biochemical indicators. Correlation networks were visualized using Cytoscape software (version 3.9.1). Statistical significance was set at *p* < 0.05 and high significance at *p* < 0.01.

## 3. Results

### 3.1. Growth Performance

**Figure 1:**
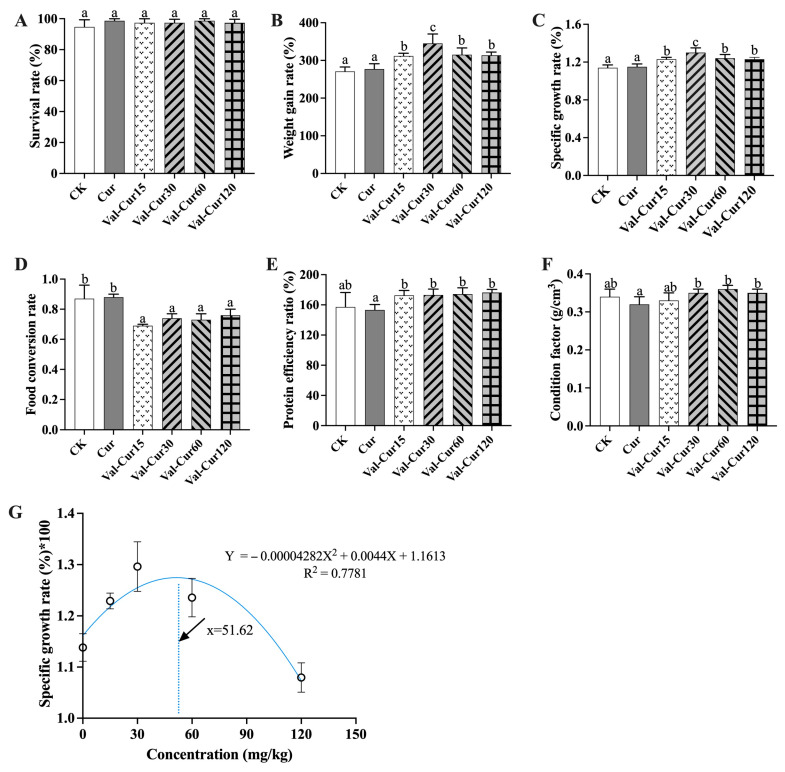
Effects of Val-Cur on growth performance of juvenile largemouth bass. (**A**) Survival rate (SR); (**B**) weight gain rate (WGR); (**C**) specific growth rate (SGR); (**D**) feed conversion rate (FCR); (**E**) protein efficiency rate (PER); (**F**) condition factor (CF); (**G**) quadratic regression of SGR. Different lowercase letters above the bars denote significant difference among groups (*p* < 0.05).

As shown in Figure 1, the SR of the fish ranged from 94.67% to 98.67% throughout the experiment, with no significant differences observed among the groups (Figure 1A). Compared to the CK group, the WGR and SGR were significantly increased in all Val-Cur-supplemented groups. The highest WGR and SGR were recorded in the Val-Cur30 group (*p* < 0.05, Figure 1B,C). Conversely, the FCR in all Val-Cur groups was significantly lower than that in the CK and Cur groups (*p* < 0.05, Figure 1D). The PER in the Val-Cur groups was higher than that in the Cur group. Moreover, the CF in the Val-Cur30, Val-Cur60, and Val-Cur120 groups was significantly greater than that in the Cur group (*p* < 0.05, Figure 1E,F). Notably, no statistically significant differences in growth performance indices were found between the Cur and CK groups (*p* > 0.05). Quadratic regression analyses based on SGR suggested an estimated optimal supplementation level of approximately 51.62 mg/kg (R^2^ = 0.778) (Figure 1G). The necessary diagnostic information for the validity of the model can be found in the Supplementary Materials.

### 3.2. Serum Biochemical Indices

**Figure 2:**
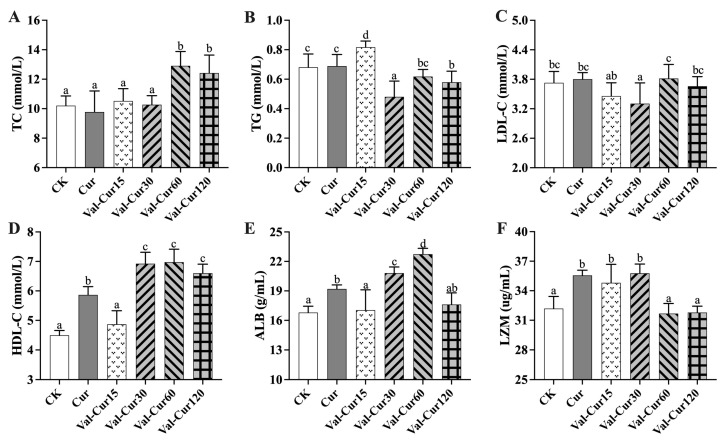
Effects of Val-Cur on serum biochemical parameters of juvenile largemouth bass. (**A**) Total cholesterol (TC); (**B**) triglyceride (TG); (**C**) low-density lipoprotein cholesterol (LDL-C); (**D**) high-density lipoprotein cholesterol (HDL-C); (**E**) albumin (ALB); (**F**) lysozyme (LZM). Different lowercase letters above the bars denote significant differences among groups (*p* < 0.05).

The results of serum biochemistry indices are shown in Figure 2. Regarding TC, TG and LDL-C, no significant differences were observed between the Cur and CK groups. Compared to the CK/Cur groups, the TG content in the Val-Cur30 and Val-Cur120 groups, as well as the LDL-C content in the Val-Cur30 group, decreased significantly (*p* < 0.05). No significant differences in TG and LDL-C were found in the other groups. However, the TC levels in the Val-Cur60 and Val-Cur120 groups and the TG level in the Val-Cur15 group were even significantly higher than those in the CK/Cur group (*p* < 0.05, Figure 2A–C). From the indicators of HDL-C, ALB and LZM, the Cur group showed significantly higher values than the CK group (*p* < 0.05, Figure 2D–F). Compared to the CK/Cur group, the levels of HDL-C in the Val-Cur30, Val-Cur60 and Val-Cur120 groups and ALB in the Val-Cur30 and Val-Cur60 groups were significantly increased. No significant differences in HDL-C and ALB were detected between the other Val-Cur groups and the CK group. In contrast, the values of these two indicators in the Val-Cur15 group were significantly lower than those in the Cur group (Figure 2D,E). As for the LZM activity, the Val-Cur15 and Val-Cur30 groups exhibited significantly higher levels than the CK group but were not significantly different from the Cur group. Conversely, the Val-Cur60 and Val-Cur120 groups showed no significant difference from the CK group but were significantly lower than the Cur group (*p* < 0.05, Figure 2F).

### 3.3. Antioxidant Enzyme Activity and Gene Expression

**Figure 3:**
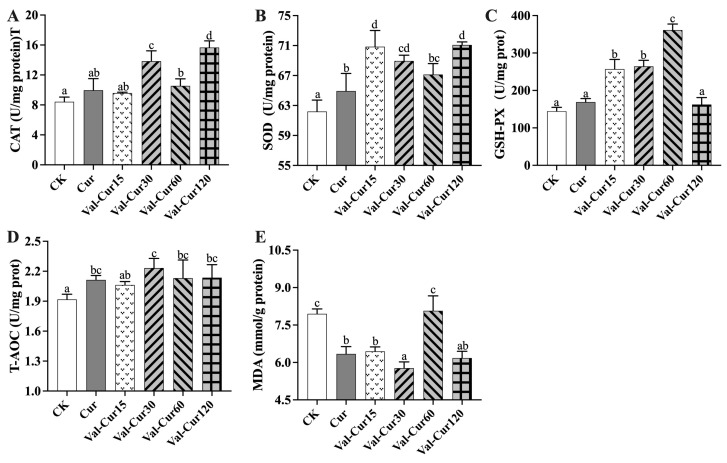
Effects of Val-Cur on antioxidant enzyme activity in juvenile largemouth bass. (**A**) Catalase (CAT); (**B**) superoxide dismutase (SOD); (**C**) glutathione peroxidase (GSH-Px); (**D**) total antioxidant capacity (T-AOC); (**E**) malondialdehyde (MDA). Different lowercase letters above the bars denote significant differences among groups (*p* < 0.05).

Compared with the CK group, SOD activity was significantly higher in all Val-Cur groups (*p* < 0.05, Figure 3B), CAT activity and T-AOC levels were increased in the Val-Cur30 to 120 groups (*p* < 0.05, Figure 3A,D), while GSH-Px activity was significantly elevated in the Val-Cur15 to 60 groups. MDA content was significantly reduced in the Val-Cur15, 30, and 120 groups (*p* < 0.05, Figure 3C,E). No significant differences in the parameters were observed between the other Val-Cur groups and the CK group. In the Cur group, SOD activity and T-AOC levels were significantly higher, and MDA content was significantly lower than in the CK group. However, CAT and GSH-Px activities did not differ significantly from the CK group (Figure 3A–C).

Compared with the Cur group, SOD activity was significantly higher in all Val-Cur groups (Figure 3B). CAT activity was significantly elevated in the Val-Cur30 to 120 groups (Figure 3A), and GSH-Px activity was significantly higher in the Val-Cur15 to 60 groups (*p* < 0.05, Figure 3C). Regarding MDA levels, the Val-Cur30 group was significantly lower, while the Val-Cur60 group was significantly higher than the Cur group (Figure 3E). No significant differences were observed in T-AOC levels between any Val-Cur group and the Cur group (Figure 3D), nor in the other antioxidant parameters not specifically mentioned above.

**Figure 4:**
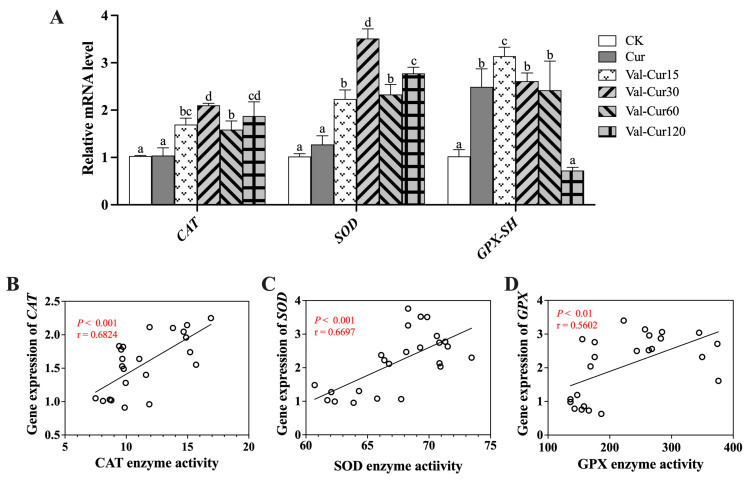
Correlation between antioxidant enzyme activities and gene expression. (**A**) Relative mRNA expression levels of antioxidant enzyme genes; (**B**–**D**) correlations between enzyme activity and gene expression for CAT, SOD and GSH-Px, respectively. Different lowercase letters above the bars denote significant differences among groups (*p* < 0.05).

The expression of antioxidant enzyme genes in the intestinal tissues was examined at the mRNA level (Figure 4). Compared with the CK group, *GPX-SH* gene expression in the Cur group was significantly higher (*p* < 0.05), whereas no significant differences were observed in *CAT* and *SOD* expression. In all Val-Cur groups, the mRNA levels of *CAT* and *SOD* were significantly up-regulated compared to the CK group (*p* < 0.05), with the Val-Cur30 group showing the highest expression. *GPX-SH* expression was also significantly increased in all Val-Cur groups except for Val-Cur120. Relative to the Cur group, the gene expression levels of *CAT* and *SOD* were significantly elevated in all Val-Cur groups. The expression of *GPX-SH* in the Val-Cur30 group was also significantly increased, while it was significantly decreased in the Val-Cur120 group. No significant differences in *GPX-SH* expression were found between the other Val-Cur groups and the Cur group.

Pearson’s correlation analysis between enzyme activities and their corresponding gene expression is shown in Figure 4B–D. The correlation coefficients for CAT, SOD, and GSH-Px with their respective gene expressions were 0.6824, 0.6697, and 0.5602, respectively, indicating strong or extremely strong positive correlations (*p* < 0.01 or *p* < 0.0001). These results suggest that the findings at the gene expression level and enzyme activity level are mutually corroborative.

### 3.4. Intestinal Morphology

**Figure 5:**
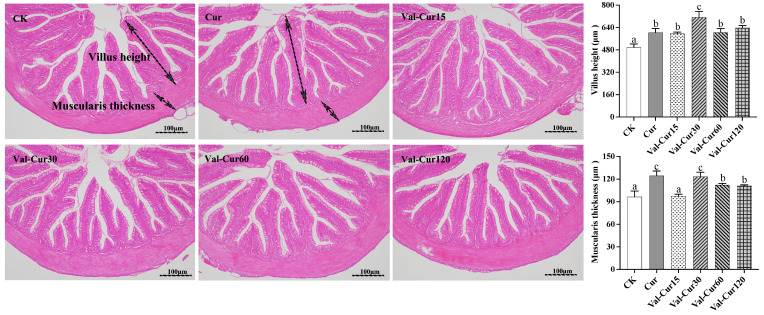
Effects of Val-Cur on the intestinal morphology of largemouth bass. Histological sections of the intestine were stained with hematoxylin and eosin (H&E) and observed at 200× magnification. Different lowercase letters above the bars denote significant differences among groups (*p* < 0.05).

Intestinal histological sections are shown in Figure 5. All groups exhibited well-aligned intestinal villi, well-preserved tissue integrity, and clearly identifiable goblet cells. Dietary supplementation with both Cur and Val-Cur significantly increased intestinal villus height and muscularis thickness in largemouth bass (*p* < 0.05). Among them, the Val-Cur30 group showed the most pronounced effect, with villus height being significantly greater than all other groups and muscularis thickness being significantly higher than the CK group and other Val-Cur supplementation groups (*p* < 0.05). The villus height in the remaining Val-Cur-supplemented groups (15, 60 and 120 mg/kg) was significantly higher than that in the CK group, but not significantly different from the Cur group. The muscularis thickness in the Val-Cur60 and Val-Cur120 groups was also significantly greater than that in the CK group, but significantly lower than that in the Cur group.

### 3.5. Expression of Genes Related to Intestinal Inflammation

**Figure 6:**
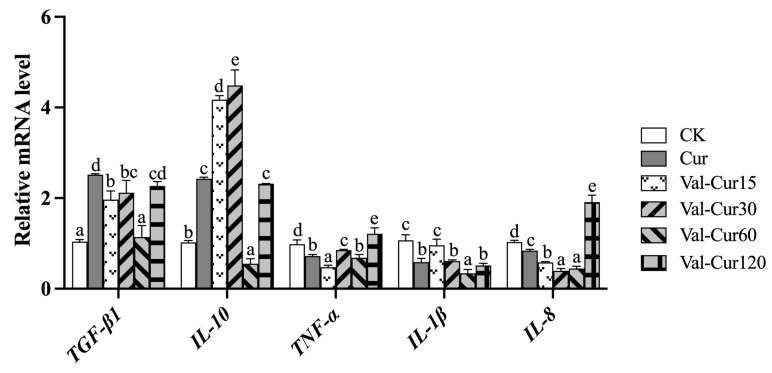
Expression of intestinal inflammation-related genes in juvenile largemouth bass. Different lowercase letters above the bars denote significant differences among groups (*p* < 0.05).

The results of the intestinal inflammatory factor expression are shown in Figure 6. Regarding the anti-inflammatory factors *TGF-β1* and *IL-10*, gene expression levels in all Val-Cur dose groups (except for Val-Cur60) and the Cur group were significantly increased compared to the CK group. Compared with the Cur group, *TGF-β1* expression was significantly decreased in the Val-Cur15 and 30 groups, while *IL-10* expression was significantly increased in these two groups. The expression of these anti-inflammatory factors in the other Val-Cur groups showed no significant difference from or was significantly lower than the Cur group.

For the pro-inflammatory factors *TNF-α*, *IL-1β* and *IL-8*, expression in the Cur group was significantly lower than in the CK group. With the exception of a few Val-Cur dose groups (e.g., *TNF-α* in Val-Cur 120, *IL-1β* in Val-Cur 15, and *IL-8* in Val-Cur 120), all Val-Cur groups also showed significantly lower expression than the CK group. Compared to the Cur group, expression levels of *TNF-α* in the Val-Cur15 group, *IL-1β* in the Val-Cur60 group, and *IL-8* in the Val-Cur15, 30, and 60 groups were significantly decreased. However, the expression levels in the remaining Val-Cur groups showed no significant difference from or were even significantly higher than for the Cur group.

### 3.6. Expression of Genes Related to Intestinal Barrier Function

**Figure 7:**
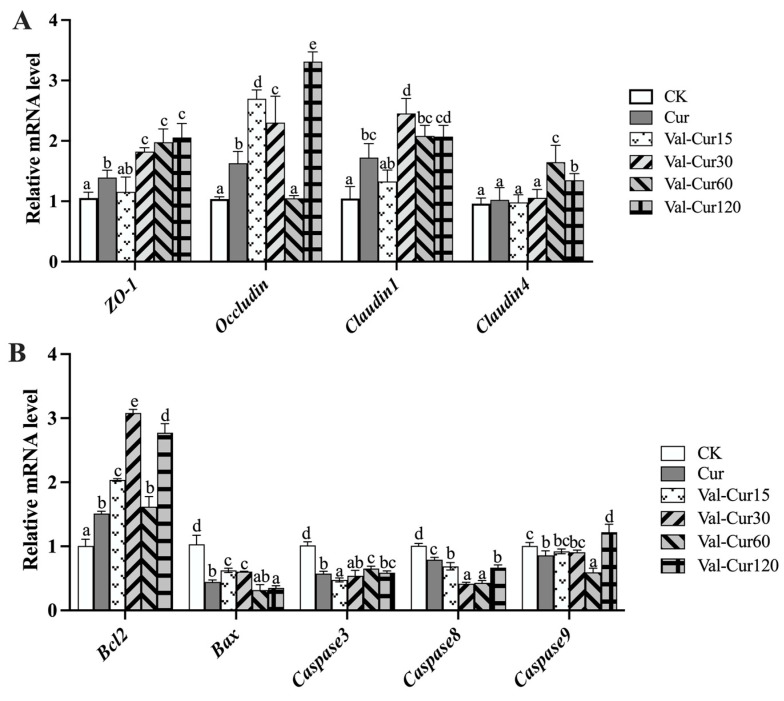
Expression levels of intestinal barrier-related (**A**) and apoptosis-related (**B**) genes in juvenile largemouth bass. Different lowercase letters above bars indicate significant differences among groups (*p* < 0.05).

The relative mRNA expression levels of genes associated with the intestinal physical barrier are shown in Figure 7A. Compared to the CK group, the Cur group showed significantly higher gene expression of all genes except for *Claudin4*. The expression of *ZO-1* in the Val-Cur30 to 120 dose groups was significantly higher than in both the CK and Cur groups. *Claudin1* expression was significantly higher in these Val-Cur groups compared to CK, and the Val-Cur30 group was also significantly higher than the Cur group. *Occludin* expression in the Val-Cur15, 30, and 120 groups was significantly higher than in both the CK and Cur groups. However, the Val-Cur60 group showed no significant difference from CK and was significantly lower than the Cur group. The expression of *Claudin4* in the Val-Cur60 and 120 groups was significantly higher than in both the CK and Cur groups (*p* < 0.05), while the other Val-Cur groups showed no significant difference from CK or Cur (Figure 7A).

Figure 7B shows the expression of the anti-apoptotic gene *Bcl2*, the pro-apoptotic gene *Bax* and the key apoptotic executor genes of the caspase family. Compared to the CK group, all Val-Cur groups and the Cur group exhibited significantly increased *Bcl2* expression, while *Bax*, and *Caspase3* and *Caspase8* expressions were significantly decreased. For *Caspase9*, the Cur and Val-Cur60 groups displayed significantly lower expression levels than CK, while the Val-Cur15 and Val-Cur30 groups showed no significant difference from CK. The Val-Cur120 group had significantly higher *Caspase9* expression than CK. Compared to the Cur group, *Bax* expression was significantly higher in the Val-Cur15 and 30 groups but lower in the Val-Cur120 group. *Caspase3* expression was significantly lower in the Val-Cur15 group but higher in the Val-Cur60 and Val-Cur120 groups (*p* < 0.05). All Val-Cur groups showed significantly lower *Caspase8* expression than the Cur group. *Caspase9* expression was significantly lower in the Val-Cur 60 group but higher in the Val-Cur 120 group compared to Cur. No significant differences were observed for the other Val-Cur groups relative to the Cur group.

### 3.7. Effects of Val-Cur on Gut Microbiota

#### 3.7.1. Alpha Diversity and Non-Metric Multidimensional Scaling Analysis

**Figure 8:**
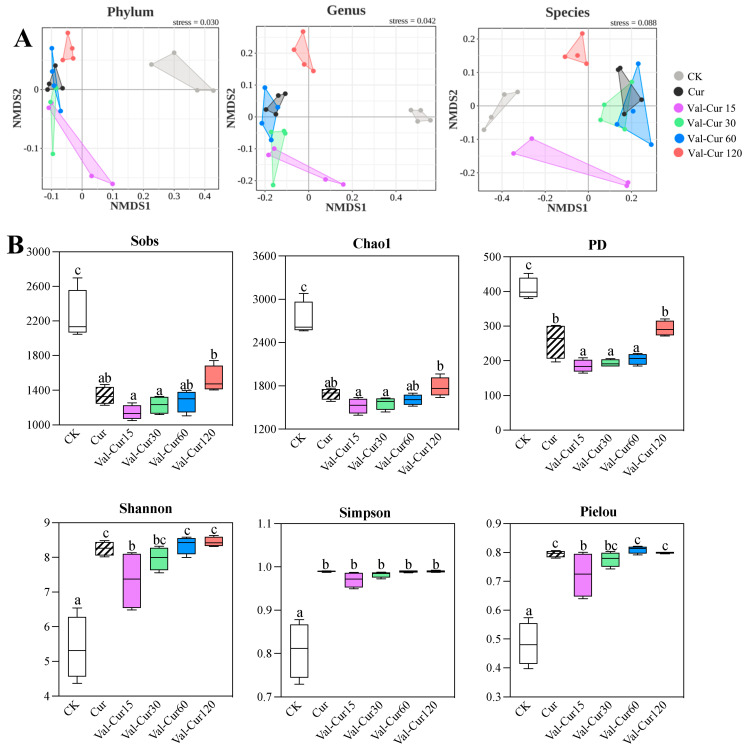
Effects of dietary supplementation on the intestinal microbial diversity of juvenile largemouth bass. (**A**) Non-metric multidimensional scaling (NMDS) plots at the phylum, genus, and species levels; (**B**) alpha diversity indices. Different lowercase letters above the bars indicate statistically significant differences (*p* < 0.05).

The results of NMDS and alpha diversity analyses of the gut microbiota are shown in Figure 8. At the phylum, genus, and species levels, samples from the CK group were clearly separated from all other groups, and samples from the Val-Cur120 group were also separated from the rest. The other groups overlapped with each other at the phylum and genus levels, but at the species level, the Val-Cur15 group was distinct from the others. This indicates significant differences in gut microbiota composition between the basal diet (CK) group and the Cur/Cur-Val supplementation group. Apart from the Val-Cur120 group, the gut microbiota composition of the two curcumin forms showed minor differences at the phylum and genus levels. The Cur, Val-Cur30, and Val-Cur60 groups exhibited similar microbial compositions at the species level, while the other groups showed considerable differences (Figure 8A). PERMANOVA based on Bray–Curtis dissimilarity revealed significant differences in microbial community composition among groups at the phylum (R^2^ = 0.7765, *p* = 0.001), genus (R^2^ = 0.6668, *p* = 0.001), and species levels (R^2^ = 0.5554, *p* = 0.001).

In terms of alpha diversity, significant changes were observed in the gut microbiota of juvenile largemouth bass in the Cur and Val-Cur groups compared to the CK group. Species richness indices (Sobs, Chao1 and PD) decreased significantly, while species diversity indices (Shannon, Simpson) and evenness index (Pielou) increased significantly (*p* < 0.05). Between the two curcumin forms, the PD index in the Val-Cur15, 30 and 60 groups were significantly lower than that in the Cur groups (*p* < 0.05). The Shannon and Pielou indices in the Val-Cur15 group were significantly lower than those in the Cur group, while no significant differences were observed between the other Val-Cur groups and the Cur group (Figure 8B).

#### 3.7.2. Intestine Microbial Composition

**Figure 9:**
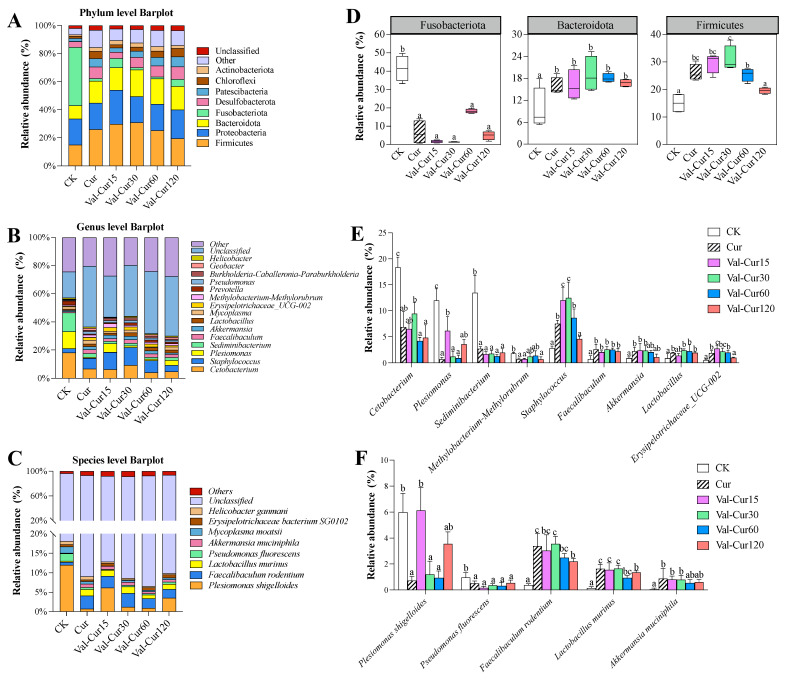
Gut microbial composition and differentially abundant taxa in juvenile largemouth bass. (**A**–**C**) Microbial composition at the phylum, genus and species levels, respectively; (**D**–**F**) relative abundance of taxa showing significant differences at the phylum, genus and species levels, respectively. Different lowercase letters above the bars indicate statistically significant difference (*p* < 0.05).

Figure 9 shows the composition of the gut microbiota in juvenile largemouth bass at the phylum, genus and species levels. A total of 37 phyla, 313 genera, and 428 species were detected. At the phylum level, Firmicutes, Proteobacteria, and Bacteroidota were the dominant bacterial taxa across all treatment groups, although their relative abundances varied among groups. The relative abundance of Firmicutes ranged from 15.05% to 31.02%, that of Proteobacteria ranged from 18.46% to 24.27%, and that of Bacteroidota ranged from 9.52% to 19.06% among the experimental groups. Other abundant phyla included Fusobacteriota, Desulfobacteriota, Patescibacteria, Chloroflexi, and Actinobacteriota (Figure 9A).

At the genus level (Figure 9B), the top 10 dominant genera were *Cetobacterium*, *Staphylococcus*, *Plesiomonas*, *Sediminibacterium*, *Faecalibaculum*, *Akkermansia*, *Lactobacillus*, and *Mycoplasma*, *Erysipelotrichaceae UCG-002* and *Methylobacterium-Methylorubrum*. At the species level (Figure 9C), the relatively dominant species (known species with abundance > 0.5%) included *Plesiomonas shigelloides*, *Faecalibaculum rodentium*, *Lactobacillus murinus*, *Pseudomonas fluorescens*, *Akkermansia muciniphila*, *Mycoplasma moatsii*, *Erysipelotrichaceae bacterium SG0102i*, and *Helicobacter ganmani*.

Compared with the CK group, Cur supplementation significantly altered the gut microbiota. At the phylum level, the abundance of Fusobacteriota decreased significantly, while Firmicutes and Bacteroidota increased significantly (Figure 9D). Among the top 10 abundant known genera, *Cetobacterium*, *Plesiomonas*, *Sediminibacterium* and *Methylobacterium-Methylorubrum* decreased significantly, whereas *Staphylococcus*, *Faecalibaculum*, *Akkermansia*, *Lactobacillus* and *Erysipelotrichaceae_UCG-002* increased significantly (Figure 9E). Among the top 15 known species, Cur treatment led to significantly decreases in *Plesiomonas shigelloides* and *Pseudomonas fluorescens*, while *Faecalibaculum rodentium*, *Lactobacillus murinus* and *Akkermansia muciniphil* increased significantly (Figure 9F).

The gut microbiota composition in the Val-Cur groups also showed significant changes compared to the CK group, with trends similar to those in the Cur group. Specifically, at the phylum level, all Val-Cur groups had a significantly reduced abundance of Fusobacteriota and increased Bacteroidota, with no significant differences from the Cur group. The Val-Cur15, 30, and 60 groups displayed significantly increased Firmicutes abundance but showed no significant difference from the Cur group. At the genus level, all the Val-Cur groups displayed significantly decreased abundance of *Cetobacterium*, *Plesiomonas*, *Sediminibacterium* and *Methylobacterium-Methylorubrum* and increased abundance of *Staphylococcus*, *Faecalibaculum*, *Akkermansia*, *Lactobacillu*, and *Erysipelotrichaceae_UCG-002*. Compared to the Cur group, *Plesiomonas* and *Erysipelotrichaceae_UCG-002* abundance was significantly higher in the Val-Cur15 group; *Staphylococcus* abundance was significantly higher in the Val-Cur15 and Val-Cur30 groups; and *Erysipelotrichaceae_UCG-002* and *Staphylococcus* abundance was significantly lower in the Val-Cur120 group. At the species level, compared to the CK group, *Plesiomonas shigelloides* abundance decreased significantly in the Val-Cur30 and Val-Cur60 groups; *Pseudomonas fluorescens* abundance decreased significantly in all Val-Cur groups; and *Faecalibaculum rodentium*, *Lactobacillus murinus* and *Akkermansia muciniphila* abundance increased (*p* < 0.05). Notably, the abundance of *Plesiomonas shigelloides* was significantly higher in the Val-Cur15 group than in the Cur group, while that of *Faecalibaculum rodentium* and *Lactobacillus murinus* was significantly lower in the Val-Cur120 group. No significant differences were observed for the remaining species compared to the Cur group (Figure 9D–F).

### 3.8. Correlation Between Gut Microbiota and Biological Indicators

**Figure 10:**
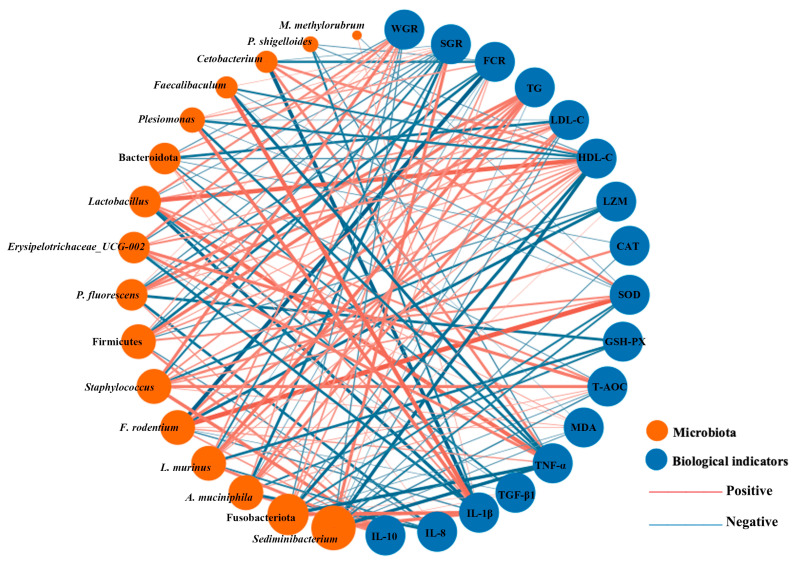
Correlation network between host biological indicators and significantly altered gut microbiota. Blue and Orange nodes represent biological indicators and bacterial taxa, respectively. Connecting edges (orange: positive correlation, blue: negative correlation) reflect Pearson correlation relationships; edge thickness corresponds to correlation strength, and node size indicates the number of connections.

To investigate the relationship between gut microbiota changes and growth performance and immune indicators, Pearson correlation analysis was conducted between 17 significantly altered microbial taxa (identified in Figure 9) and indicators of growth performance (WGR, SGR and FCR), serum biochemistry (TC, TG, LDL-C, HDL-C, ALB, LZM), antioxidant enzyme activities (CAT, SOD, GSH-Px, T-AOC, MDA), and inflammatory factors (*TNF-α*, *IL-8*, *IL-1β*, *TGF-β1*, *IL-10*). These results are shown in Figure 10.

Sixteen of the seventeen bacterial taxa showed significant correlations with growth performance and serum biochemical indicators. Specifically, Firmicutes, Bacteroidota, *Staphylococcus*, *Lactobacillus*, *Faecalibaculum*, *Erysipelotrichaceae_UCG-002*, *Faecalibacterium rodentium*, *Lactobacillus murinus* and *Akkermansia muciniphila* were positively correlated with growth performance (WGR, SGR), serum biochemistry (HDL-C and LZM), antioxidant enzyme activities (CAT, SOD, GSH-Px and T-AOC), and anti-inflammatory factors (*TGF-β1*, *IL-10*), while being negatively correlated with FCR, TG, LDL-C, MDA, and pro-inflammatory factors (*TNF-α*, *IL-8, IL-1β*). In contrast, Fusobacteriota, *Cetobacterium*, *Plesiomona*, *Sediminibacterium*, and *Pseudomonas fluorescens* were significantly negatively correlated with growth performance (WGR and SGR), serum biochemistry (HDL-C, LZM), antioxidant enzyme activities (CAT, SOD, GSH-Px, T-AOC), and the anti-inflammatory factor *TGF-β1*, but positively correlated with FCR, TG, LDL-C, MDA, and pro-inflammatory factors (*TNF-α*, *IL-1β*). Additionally, *Methylobacterium-Methylorubrum* was negatively correlated with *TGF-β1* and positively correlated with *IL-1β. Plesiomonas shigelloides* showed significant negative correlations with LDL-C, HDL-C, and T-AOC, and a significant positive correlation with *IL-1β*.

## 4. Discussion

### 4.1. Val-Cur Enhances Growth Performance and Serum Immunity More Effectively

Previous studies have reported inconsistent effects of dietary Cur supplementation on the growth performance of juvenile largemouth bass. For instance, supplementation of 60 mg/kg Cur was shown to increase the WGR and SGR, while reducing the FCR [12]. In contrast, the addition of 0.1–0.2% (1000–2000 mg/kg) nano-curcumin did not significantly promote growth [16]. Another study indicated that although a high dose of 10 g/kg (10,000 mg/kg) Cur increased feed intake and growth in largemouth bass, it had no significant effect on FCR, viscerosomatic index, or hepatosomatic index [17]. These discrepancies may be attributed to variations in experimental conditions (e.g., fish size, rearing environment) and the inherently low bioavailability and instability activity of Cur. In the present study, although 60 mg/kg Cur did not significantly improve growth, all tested doses of water-soluble Val-Cur resulted in significantly better growth performance than the Cur group. Moreover, the optimal dietary Val-Cur level for promoting growth was determined to be 51.62 mg/kg, which is substantially lower than the effective Cur doses reported in the literature (ranging from 60 mg/kg to 20 g/kg). This highlights the advantages of Val-Cur in terms of application dosage and growth-promoting efficiency. Furthermore, Cur has been documented to significantly modulate serum biochemical parameters in fish. Dietary Cur supplementation (200–5000 mg/kg) effectively improved lipid metabolism, significantly reducing serum or hepatic levels of TC, TG and LDL-C in American eel [18], Marbled eel [19], and large yellow croaker fed a high-fat diet, while increasing HDL-C levels and enhancing intestinal antioxidant enzyme (CAT, SOD, GSH-Px) activities [20]. Cur supplementation (60–393.67 mg/kg) also increased serum ALB levels in juvenile largemouth bass and enhanced serum LZM activity in tilapia [21], grass carp [22], yellow catfish [23], and American eel [18]. Our findings confirm that 60 mg/kg Cur, as well as 15–30 mg/kg Val-Cur, can replicate these physiological improvements. Notably, however, a dose of just 30 mg/kg Val-Cur was significantly more effective than 60 mg/kg Cur in regulating blood lipids, specifically by lowering LDL-C and elevating HDL-C.

### 4.2. Val-Cur Exerts Superior Anti-Inflammatory Effects

The key to intestinal inflammatory response lies in the dynamic balance between pro-inflammatory factors (*TNF-α*, *IL-8*, and *IL-1β*) and anti-inflammatory factors (*TGF-β1* and *IL-10*) [24]. Previous studies have found that Cur can alleviate inflammation in fish through the bidirectional regulation of these cytokines. For instance, 45 μmol/L Cur suppressed the expression of *TNF-α* and *IL-1β* in yellow catfish cells under high ammonia nitrogen stress [25]. Dietary Cur at 200–400 mg/kg downregulated the levels of *TNF-α*, *IL-8* and *IL-1β* in the liver and spleen of bighead carp, mitigating pesticide-induced stress [26]. Furthermore, a high dose of 10–15 g/kg Cur not only significantly reduced the mRNA levels of pro-inflammatory factors in the liver of common carp but also significantly upregulated the expression of anti-inflammatory factor *IL-10* [27]. Even 35 mg/kg of nano-curcumin enhanced *IL-10* expression in tilapia, suggesting that formulation improvements can enhance its bioactivity [28]. In the present study, 60 mg/kg Cur also exhibited significant anti-inflammatory effects: it significantly downregulated the intestinal mRNA levels of *TNF-α*, *IL-8* and *IL-1β*, while promoting the expression of *TGF-β1* and *IL-10* in juvenile largemouth bass. Notably, a dose of just 30 mg/kg Val-Cur achieved comparable suppression of pro-inflammatory factors and variably promoted the expression of anti-inflammatory factors. Moreover, the upregulation of *IL-10* in the low-dose Val-Cur groups (15–30 mg/kg) was significantly greater than that in the 60 mg/kg Cur group. The enhanced efficacy of Val-Cur observed in the present study may be related to its improved physicochemical characteristics compared with Cur. Previous studies have shown that increasing the solubility and bioavailability of curcumin can substantially improve its biological activity [10]. Therefore, the superior performance of low-dose Val-Cur may be attributed to enhanced intestinal absorption and greater systemic availability, allowing more efficient regulation of antioxidant and immune-related pathways (like inhibition of the NF-κB signaling pathway, which controls the transcription of pro-inflammatory cytokines such as *TNF-α*, *IL-1β*, and *IL-8*).

### 4.3. Val-Cur Improves Intestinal Barrier Function at Lower Doses

A healthy intestinal structure, characterized by villus height and muscularis thickness, forms the foundation for nutrient absorption and physical barrier function [29]. This study found that both Cur and Val-Cur significantly increased intestinal villus height and muscularis thickness in juvenile largemouth bass, with the most pronounced effects observed in the Val-Cur30 group. This aligns with findings in marbled eel [19], American eel [18], tilapia [30], and large yellow croaker [31], where Cur supplementation (30–300 mg/kg) similarly improved intestinal morphology. Furthermore, intestinal barrier homeostasis relies on the regulation of tight junction protein expression and apoptotic balance. Studies have shown that 400 mg/kg Cur alleviates toxin-induced damage to the physical barrier in the gills of grass carp and the intestine of wild duck by upregulating tight junction proteins (*Zo-1*, *Occludin*, and *Claudin-1*) and inhibiting apoptosis pathways (Caspase3/8/9) [32,33]. A similar protective mechanism via Cur was observed in mouse intestinal injury caused by bacterial endotoxin [34]. The present study confirmed that 60 mg/kg Cur enhanced the mRNA expression of *ZO-1*, *Occludin*, and *Claudin-1* genes in largemouth bass, while increasing the expression of the anti-apoptotic factor *Bcl-2* and decreasing the expression of pro-apoptotic factors (*Bax* and *Caspase3/8/9*). Notably, Val-Cur at lower doses (15–30 mg/kg) demonstrated comparable or superior regulatory effects to 60 mg/kg Cur: its promotion of tight junction proteins was comparable to the Cur group in the Val-Cur15 group and stronger in the Val-Cur30 group, while its regulation of apoptosis factors (e.g., increasing *Bcl-2* and decreasing *Caspase8*) showed a stronger trend. In summary, Val-Cur promotes the growth of juvenile largemouth bass at lower doses by improving intestinal morphology, enhancing barrier protein expression, and regulating cell apoptosis.

### 4.4. Val-Cur Beneficially Modulates the Gut Microbiota

The gut microbiota of fish is extensively involved in various biological processes of the host, including nutrition metabolism, intestinal differentiation, and immune defense, thereby maintaining host health [35]. In this study, both forms of curcumin reduced the alpha diversity of the gut microbiota but increased its evenness. This phenomenon is consistent with findings in American eels [18], marbled eel [19], and abalone [8]. The reduction in gut microbiota diversity by curcumin may be related to its antibacterial properties. However, further analysis revealed that the reduced relative abundances primarily involved potentially harmful bacteria. For instance, the abundance of common aquaculture pathogens such as *Plesiomonas*, *Plesiomonas shigelloides*, and *Pseudomonas fluorescens* decreased significantly in the Cur and Val-Cur groups. *Plesiomonas* and *Plesiomonas shigelloides* can cause zoonotic gastrointestinal diseases [10]. *Pseudomonas fluorescens* can lead to pseudomoniasis in fish and shrimp, typically characterized by body ulcers, septicemia, petechial hemorrhages, skin darkening, scale loss, ascites and popeye disease [36,37]. *Methylobacterium-Methylorubrum* and *Sediminibacterium* are considered bacteria associated with disease biomarkers and can infect immunocompromised fish [38,39].

Conversely, Cur/Val-Cur treatment significantly increased the abundance of some potentially probiotic bacterial groups. The significantly increased phyla, Firmicutes and Bacteroidetes, are typically producers of short-chain fatty acids (SCFAs), particularly butyrate. Butyrate, an end product of colonic fermentation, plays a crucial role in maintaining intestinal health [40]. *Staphylococcus*, *Erysipelotrichaceae_UCG-002*, *Akkermansia*, *Faecalibaculum*, and *Lactobacillus* can also exert anti-inflammatory effects by producing SCFAs (e.g., acetate, propionate, butyrate, and lactate) [41,42,43]. *Akkermansia muciniphila* exhibits benefical probiotic activity against many metabolism-related diseases, such as obesity, diabetes, and inflammatory bowel disease [44,45]. This bacterium can also ameliorate high-fat-diet-induced hepatic steatosis and enteritis in zebrafish [46]. *Faecalibaculum rodentium* exerts anti-tumor and anti-inflammatory effects through SCFA production [47]. *Lactobacillus murinus* is a common probiotic that can inhibit inflammation and regulate apoptosis by modulating the NF-κB/MAPK signaling pathway [48,49]. The reduction in microbial diversity observed in this study may indicate a shift from a heterogeneous community to a more functionally specialized microbiota, driven by dietary modulation. This process likely reflects the enrichment of beneficial taxa and suppression of opportunistic bacteria, resulting in improved host-microbe interactions and enhanced intestinal homeostasis.

### 4.5. Val-Cur Acts Synergistically with Microbiota to Enhance Health

In this study, the changes in gut microbiota induced by Cur and Val-Cur were significantly correlated with improvement in physiological indicators. *Plesiomonas shigelloides* and *Pseudomonas fluorescens* can easily cause sepsis, pseudomoniasis, and other aquaculture diseases [50,51]. *Methylobacterium-Methylorubrum* and *Sediminibacterium* readily infect immunocompromised fish. The reduction in abundance of these bacteria showed a significant positive correlation with increased expression of the pro-inflammatory cytokine IL-1β. In contrast, the enrichment of some probiotic groups was positively correlated with improvements in growth performance, blood lipid profiles, and immune parameters. These probiotics include Firmicutes, Bacteroidota, *Erysipelotrichaceae_UCG-002*, *Faecalibaculum rodentiu*, *Lactobacillus murinusm*, and *Akkermansia muciniphila*, all of which can produce SCFAs. SCFAs can inhibit inflammation by suppressing fat absorption and improving insulin sensitivity [52,53]. Some beneficial bacterial species of Lactobacillus are often supplemented into aquafeeds as antibiotic alternatives, promoting growth, enhancing serum enzyme activity, and improving immunity in common carp [54], rainbow trout [55], and largemouth bass [56]. The results of this study demonstrate that these probiotics show significant positive correlations with enhanced growth performance (↑WGR and SGR), improved serum biochemical indices (↑HDL-C and LZM), and increased antioxidant capacity (↑CAT, SOD, GSH-Px, and T-AOC) in juvenile largemouth bass.

Overall, the present study demonstrated the beneficial effects of Val-Cur in aquaculture. However, the highest supplementation level (120 mg/kg) did not consistently produce greater benefits than lower doses, and certain responses (such as increased *Caspase9* expression and TC levels), suggested that the effects of Val-Cur may not increase linearly with dosage. Therefore, further dose–response and long-term safety studies are required to determine the optimal supplementation level and upper safety limit of Val-Cur in aquafeeds. Although Val-Cur was incorporated into the feed matrix during pellet production and rapidly consumed by fish after feeding, the potential leaching of this highly water-soluble compound was not directly quantified in water or residual feed. Our results should be interpreted as responses to nominal dietary concentrations rather than as exact delivered doses. Future studies incorporating direct chemical measurements would further strengthen exposure validation. Moreover, Val-Cur significantly altered the intestinal microbial community structure, and the resulting shifts in microbial composition may have influenced the metabolism and bioactivity of curcumin-derived compounds. Previous studies have shown that gut bacteria can convert curcumin to tetrahydrocurcumin and other metabolites with distinct biological activities [57]. Therefore, the physiological effects observed in this study may be partly mediated by microbiota-dependent metabolism. Future studies integrating leaching assessments, metabolomic analyses, and functional validation are needed to further elucidate the mechanisms underlying the effects of Val-Cur in aquatic animals.

## 5. Conclusions

This study demonstrates that water-soluble curcumin (Val-Cur), at a significantly lower dietary dose (15–30 mg/kg) than Cur (60 mg/kg), more effectively promotes growth, improves serum biochemical profiles, and enhances intestinal antioxidant and anti-inflammatory capacities in juvenile largemouth bass. The underlying mechanisms involve a synergistic effect whereby Val-Cur remodels the gut microbiota (inhibiting potential pathogens and enriching beneficial bacteria), strengthens the intestinal physical barrier, and modulates the expression of genes related to immunity and apoptosis, ultimately enhancing the overall health and growth performance of the host. Notably, the highest dose (120 mg/kg) did not confer additional benefits and showed a non-linear dose–response pattern, with elevated *Caspase9* and TC at this level warranting further safety evaluation. The high-water solubility and bioavailability of Val-Cur are key to its high efficacy at low application doses.

## Figures and Tables

**Table 1:** Primer sequences for real-time quantitative PCR.

Primers	Sequences (5′–3′)	Accession Number
*β*-*actin*-F	5′-GGACACGGAAAGGATTGACAG-3′	XM038695351.1
*β*-*actin*-R	5′-CGGAGTCTCGTTCGTTATCGG-3′	
*CAT*-F	5′-GTTCCCGTCCTTCATCCACT-3′	MK614708.1
*CAT*-R	5′-CAGGCTCCAGAAGTCCCACA-3′	
*SOD*-F	5′-CTGACCTACGACTATGGTGC-3′	MK64709.1
*SOD*-R	5′-CGTCACATCTCCCTTCGCTA-3′	
*GPX_1a*-F	5′-CCCTGCAATCAGTTTGGACA-3′	MK64713.1
*GPX_1a*-R	5′-TTGGTTCAAAGCCATTCCCT-3′	
*TNF-α-F*	5′-GCAGCAGCAGTGATGATGATGAC-3′	XM038723994.1
*TNF-α-R*	5′-AGGATGGTCTGGTACGACTTGTTG-3′	
*TGF-β1-F*	5′-TCATCCGCACGCTCAACTATCC-3′	XM038712764
*TGF-β1-R*	5′-GTGCTCTGGCTGTTGGAGTAGG-3′	
*IL-8-F*	5′-TCCTGGCTGCTCTGGCTCTC-3′	XM038704089
*IL-8-R*	5′-GGAGAAGAGGTCGTCCGTATGC-3′	
*IL-10-F*	5′-AGCAGCATCATTACCACTGAGGAC-3′	XM038696252
*IL-10-R*	5′-AACCAGGACGGACAGGAGGAG-3′	
*IL-1β-F*	5′-ACAGCCTGGTGGACGAAACG-3′	XM038733429
*IL-1β-R*	5′-TGCGGTCGCTCAGAGTGATTG-3′	
*Zo-1*-F	5′-TACAACCAGGATTCTCACCTG-3′	XM038701018.1
*Zo-1*-R	5′-TTGTTCTCAAACATTTTGACCCTAG-3′	
*Occludin*-F	5′-CTGGTCGTCGTCGCTCTCATC-3′	XM038734217
*Occludin*-R	5′-TGTTGCTCTTGCCGAACTCCTG-3′	
*Caludin-1*-F	5′-AATTCGGAAGTGCCCTGTTTGTTG-3′	XM038718401
*Caludin-1*-R	5′-TGTTGCTCTTGCCGAACTCCTG-3′	
*Caludin-4*-F	5′-GGGAGGGTTTGTGGATGGACTG-3′	XM038707645.1
*Caludin-4*-R	5′-CAGCGATAATGGCGACGATGATG-3′	
*Bax*-F	5′-GCAGCAGCAGTGATGATGATGAC-3′	XM038704178.1
*Bax*-R	5′-AGGATGGTCTGGTACGACTTGTTG-3′	
*Bcl*-F	5′-TCATCCGCACGCTCAACTATCC-3′	XM038711460.1
*Bcl*-R	5′-GTGCTCTGGCTGTTGGAGTAGG-3′	
*Caspase-3*-F	5′-GCCGTGGTACAGACCTGGATG-3′	XM038699323.1
*Caspase-3*-R	5′-AGCCTGGAGCAGTGGAATAAGC-3′	
*Caspase-8*-F	5′-GGGACAAAGAGGTGGAGGAAGAC-3′	XM038718637.1
*Caspase-8*-R	5′-GGATGTAGATGGAGCCTGTGGAAG-3′	
*Caspase-9*-F	5′-AGACGGGTCAGCACAGTTTGG-3′	XM038722308.1
*Caspase-9*-R	5′-GGCAAGACAACAGGGTGAACAAC-3′	

## Data Availability

The data that support the findings of this study are available from the corresponding author upon reasonable request.

## Supplementary Materials

The following supporting information can be downloaded at: https://www.mdpi.com/article/10.3390/ani16132032/s1, Table S1. The full quadratic regression output. Figure S1. (**A**) Residuals vs. Fitted Values; (**B**) Normal Q–Q Plot of Residual.
