# Supplementary material for: Valine-Curcumin Improves Growth, Intestinal Immunity, and Microbiota in Largemouth Bass (Micropterus salmoides)
Source: Animals (Basel). 2026 Jul 2;16(13):2032. doi: 10.3390/ani16132032 (PMC13359457; doi:10.3390/ani16132032)
Supplement: Supplementary file 1 [file animals-16-02032-s001.zip › animals-4378308-supplementary.pdf]

Valine-Curcumin Improves Growth, Intestinal Immunity, and Microbiota in Largemouth Bass (*Micropterus salmoides*)

Jing Ni [1], Hejian Xiong [2], Ruifang Wang [2], Yuanhong Xie [2], Lixing Huang [1], Ying Ma [1*], Chuanbo He [2*]

[1] Fisheries college of Jimei University, State Key Laboratory of Mariculture Breeding, Xiamen 361021, China
[2] College of Ocean Food and Biological Engineering, Jimei University, Xiamen 361021, China
* Correspondence: Ying Ma, Email: maying@jmu.edu.cn; Chuanbo He, Email: hcbcc@jmu.edu.cn

Full regression outputs for the dose-response model

Supplementary Table S1

Table S1. The full quadratic regression output

Parameter	Estimate	Std.Error	95% (CI)	*P*-values
Intercept (A)	1.1613	0.0211	1.1153 - 1.2072	< 0.001
Linear (B)	0.00441	0.00100	0.00224 - 0.00658	0.001
Quadratic (C)	-0.0000428	0.0000078	-0.00006 - -0.000026	< 0.001

The model showed good explanatory power ($R^2 = 0.778$; adjusted $R^2 = 0.741$), with an overall model significance of $P = 0.00012$. The estimated optimal dietary Val-Cur level was 51.62 mg/kg.

Supplementary Figures

Regression diagnostic plots were generated based on the fitted quadratic model and are presented in Supplementary Figure S1A–B. The residuals-versus-fitted values plot was used to assess model fit and variance homogeneity, whereas the normal Q–Q plot was used to evaluate residual normality. The residuals were randomly distributed around zero with no obvious pattern suggesting heteroscedasticity or model misspecification. In addition, the Q–Q plot showed no substantial deviation from normality. Together, these diagnostic results support the adequacy of the fitted quadratic model.

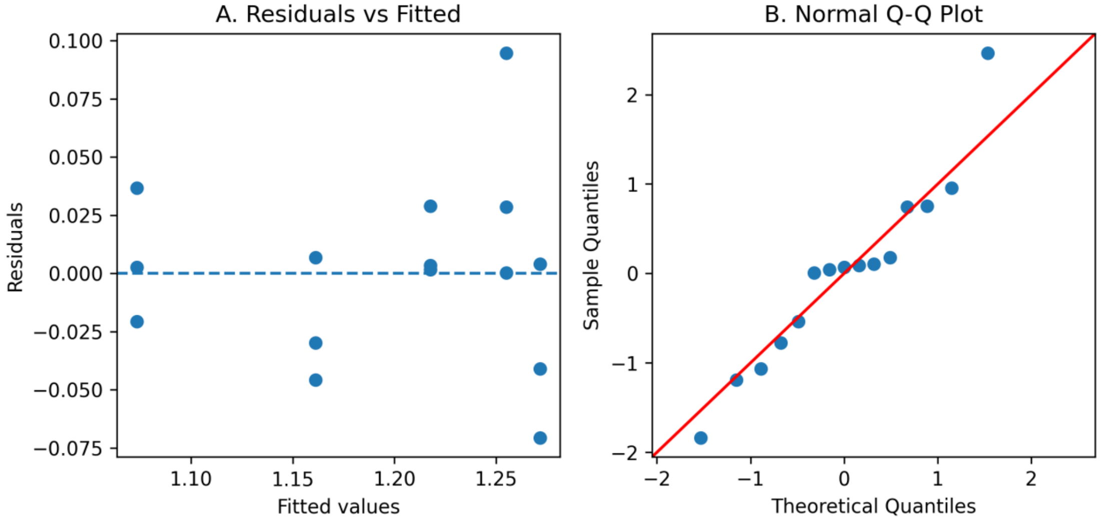

Figure. S1. (A) Residuals vs Fitted Values; (B) Normal Q－Q Plot of Residual.
